# Changes in Endotracheal Cuff Pressure During Positioning From Supine to Beach Chair in Shoulder Surgery: A Prospective Case Series

**DOI:** 10.7759/cureus.86660

**Published:** 2025-06-24

**Authors:** Elena Garrido, Andrew Bisenius, Liem Nguyen, Truc Nguyen, Elyana Wohl, Yasser El-Hattab, Henry T Hoffman, Franklin Dexter

**Affiliations:** 1 Department of Anesthesia, University of Iowa, Iowa City, USA; 2 Department of Otolaryngology - Head and Neck Surgery, University of Iowa, Iowa City, USA

**Keywords:** beach chair position, complication, endotracheal cuff pressure, monitoring, pressure manometer

## Abstract

Introduction

Changes in a patient’s position from supine to upright may alter the forces exerted by the endotracheal tube (ETT) on the larynx and potentially increase intracuff pressure. This study aimed to investigate whether positional changes from the supine to the beach chair position lead to an increase in intracuff pressure. Additionally, we examined whether the intracuff pressure, when the cuff is inflated using an air syringe, is within the recommended safe range (20-30 cm H₂O) both in the supine position after intubation and after transitioning to the beach chair position.

Case presentation

This prospective case series enrolled nine patients scheduled for elective shoulder surgery in the beach chair position between July 1 and 31, 2024. All patients received general anesthesia, and tracheal intubation was performed using a high-volume, low-pressure cuff ETT. Immediately after intubation, the ETT cuff was inflated using a syringe with air, guided by manual palpation of the pilot. A member of the research team measured the cuff pressure using a manometer following the initial inflation and again after the patient was repositioned into the beach chair position, immediately before surgical prepping. In both instances, the pressure was adjusted to 30 cm H₂O. Data are reported as median (25th percentile, 75th percentile).

Results

The median age of the patients was 65 (61, 75) years. Initial intracuff pressures exceeded 30 cm H₂O in all nine patients, with a mean of 73 cm H₂O and a median of 60 cm H₂O (60, 100). After adjustment of the cuff pressure to 30 cm H₂O and the patient seated up, the mean intracuff pressure did not differ significantly from 30 cm H₂O (99% CI 21-46, p = 0.35).

Conclusion

Changing the patient’s position from supine to beach chair had a negligible effect on intracuff pressure. Similar to earlier studies, we observed that using a syringe and manual palpation often resulted in cuff overinflation (>30 cm H₂O) in many patients. We recommend the routine use of a manometer to check ETT cuff pressure, particularly in prolonged cases, to reduce the risk of laryngeal complications.

## Introduction

The beach chair position is commonly employed for upper limb procedures. However, in this position, stabilizing the patient’s head can be challenging [1]. Changes in patient positioning may alter the forces exerted by the endotracheal tube (ETT) on the larynx [2]. Kako et al. reported that variations in head and neck position from supine to upright may change the shape and length of the trachea, as well as the position of the cuff, in the pediatric population, resulting in alterations in intracuff pressure [3]. In adult patients, changing from a 35^◦^semi-Fowler position to the lateral decubitus position can also cause significant variations in cuff pressure [4]. ETT intracuff pressure should be maintained within the recommended safe ranges (20-30 cm H₂O), as pressures below 20 cm H₂O may allow micro-aspiration of the oropharyngeal contents, increasing the risk of ventilator-associated pneumonia [5], while pressures above 30 cm H₂O can damage the tracheal mucosa [6].

In anterior cervical spine surgery (ACSS), an increase in ETT pressure following retractor placement has been implicated as a possible cause of postoperative vocal fold paralysis [7]. When the ETT is fixed at the mouth with tape and within the larynx or trachea by an overinflated cuff, the tube becomes rigidly positioned, potentially compressing the larynx and the recurrent laryngeal nerve [8]. Kriskovich et al. identified vocal fold paralysis consistent with recurrent laryngeal nerve injury in 3% of cases (n = 30) from a large series of 900 patients undergoing ACSS [9]. This was attributed to intraoperative increases in cuff pressure and reduced electromyographic activity, likely resulting from the interaction between retractor placement and the ETT [9]. Laryngeal electromyography plays a key role in assessing the severity of nerve damage and predicting the prognosis of nerve recovery [10].

Rubio-Nazabal et al. reported a case of bilateral recurrent laryngeal nerve compression associated with unnoticed and prolonged overinflation of the endotracheal cuff resting just below the vocal cords. In their literature review, they identified the beach chair position as a possible contributing factor [11]. Similarly, authors of a case report on arytenoid cartilage dislocation following shoulder surgery emphasized the importance of monitoring neck position and checking intracuff pressure in patients undergoing procedures in the beach chair position [2].

Endotracheal cuff pressures are not routinely measured, and several studies have demonstrated that palpation of the pilot balloon is an unreliable method for detecting high cuff pressures [12]. Given that previous research has demonstrated significant variations in cuff pressure due to changes in body position [4], we aimed to compare ETT cuff pressure shortly after intubation in the supine position and after repositioning to the beach chair position during shoulder surgery, a comparison that has not been previously investigated.

The primary aim of this study was to determine whether changing the patient's position from supine to beach chair position increases intracuff pressure, thereby potentially increasing the risk of postoperative laryngeal complications. Additionally, we aimed to assess whether cuff pressure following inflation using an air syringe and palpation method falls within the recommended safe range of 20-30 cm H₂O.

## Materials and methods

Case presentation

This prospective case series was conducted from July 1-31, 2024, in the Department of Anesthesia at the University of Iowa Hospitals and Clinics. Approval was obtained from the Institutional Review Board of the University of Iowa (approval number: 202303455).

Inclusion criteria were patients aged 18 years or older, scheduled for elective shoulder surgery in the beach chair position, use of an ETT with a high-volume, low-pressure cuff, ETT size 7.0 for women and 7.5 for men, intraoperative access to the pilot balloon, and English-speaking, with written informed consent for participation. Exclusion criteria included a history of difficult intubation and/or baseline dysphonia and ETT size other than 7.0 for women or 7.5 for men.

All surgeries were performed by the same orthopedic surgeon who specializes in arthroscopic shoulder surgery. At our institution, three orthopedic surgeons typically perform these processes. However, to minimize variability due to differences in surgical technique, we chose to include only cases performed by a single surgeon throughout the study.

During the study period, nine consecutive patients scheduled for shoulder surgery with this surgeon were invited to participate. All nine agreed to enroll, and all were included in the study.

The type of anesthesia was determined by the attending anesthesia team assigned to each case, with no specific protocol mandated for the study.

Procedure

Immediately after intubation, the circulating nurse or a member of the anesthesia care team assigned to the case inflated the ETT cuff using the standard method (typically syringe inflation and pilot balloon palpation). The anesthesiologist who performed the intubation did not inflate the cuff. Within five minutes, a member of the research team connected a pressure manometer (Posey 8199 Cufflator Endotracheal Tube Inflator and Manometer, Posey, Arcadia, CA, USA) to the ETT’s pilot balloon, and the cuff pressure was adjusted to a range of 28-30 cm H₂O. This was defined as the “initial pressure supine.” Once the patient was positioned in the beach chair position by the surgeon and immediately before prepping, the cuff pressure was checked again using the same pressure manometer by a member of the research team. This reading was defined as the “pressure sitting before prepping.” If there was a change in pressure, it was readjusted to between 28 and 30 cm H₂O using the Posey 8199 Cufflator Endotracheal Tube Inflator and Manometer. All manometer readings were recorded.

Statistical analysis

The initial pressure, the pressure sitting before prepping, and the pressure difference were considered the dependent variables. The demographic variables included age, sex, BMI, number of intubation attempts, and the device used for intubation (direct laryngoscope or video laryngoscope). One-group Student's t-tests were used for each dependent variable. A p-value < 0.01 was considered statistically significant, and 99% CIs were reported. Each demographic variable was compared with each dependent variable using Spearman rank correlations, with exact p‑values calculated using 10,000 Monte Carlo permutations (Stata v18.5 spearman command; StataCorp LLC, College Station, TX, US).

According to Fritz et al. [13], a change of 11 cm H₂O from a baseline of 28 cm H₂O was associated with sore throat and hoarseness. Based on data from Kim et al. [14], a standard deviation of 6.7 cm H₂O may be expected when changing positions. Using a significance level of p < 0.01, the study had 90% statistical power to detect a difference of 11 cm H₂O, if present.

## Results

Nine patients were included in the study (4 women and 5 men).

No significant associations were found between patient or case characteristics (Table 1).

**Table 1:** Demographic data Adjustment for multiple comparisons to control the false discovery rate was not performed because none of the associations were statistically significant (p < 0.005), even without adjustment. The reported p-values reflect comparisons of the observed Spearman rho values against a null hypothesis of zero correlation.

		Initial pressure supine		Pressure sitting before prepping		Difference	
	Median (IQR)	Spearman rho	Exact p-value	Spearman rho	Exact p-value	Spearman rho	Exact p-value
Age	65 (61, 75)	0.29	0.45	0.19	0.92	-0.46	0.21
BMI	32.88 (28.25, 35.7)	0.33	0.37	-0.72	0.62	-0.21	0.57
Female (%)	44% (4/9)	N/A	0.71	N/A	0.60	N/A	0.41
Number of attempts	1 (1, 1)	0.17	1.0	0.13	0.05	0.00	0.88
Direct laryngoscope	67% (6/9)	0.03	0.16	N/A	0.19	N/A	0.38

The initial cuff pressures exceeded 30 cm H₂O in all nine patients, with a mean of 73 cm H₂O and a median of 60 cm H₂O (60, 100). After adjustment of the cuff pressure to 30 cm H₂O and the patient seated up, the mean pressure did not differ significantly from 30 cm H₂O (99% CI 21-46, p = 0.35) (Table 2).

**Table 2:** Changes in endotracheal tube cuff pressure at supine and after changing position from supine to beach chair *p < 0.001.

	Initial pressure	Pressure sitting before prepping	Pressure difference
Median (IQR)	60 (60, 100)	32 (25, 40)	-40 (-50, -18)
Mean (standard deviation)	72.6 (27.5)	33.6 (-11.1)	-39.0 (25.4)
Median of difference from 30 cm H₂O (25th percentile, 75th percentile)	30 (30, 70)	2 (-5, 10)	N/A
Mean of difference from 30 cm H₂O (standard deviation)	42.6 (27.5)	3.6 (11.1)	N/A
One-group Student's t-test (two-sided) p-value comparing mean to 30 cm H₂O	0.0017*	0.35	N/A
One-group Student's t-test (two-sided) p-value comparing mean to 0 cm H₂O	N/A	N/A	0.0018*

## Discussion

In our study, we observed that changes in the patient’s position from supine to sitting did not systematically alter the mean intracuff pressure. Postural changes and associated head and neck movements can displace the ETT and modify intracuff pressure [4]. For example, Kako et al. identified ETT movement associated with changes in head and neck position as a major cause of cuff overinflation [3]. Similarly, another study observed that intracuff pressure increased significantly during neck flexion, both in the supine and prone positions [3]. Minonishi et al. also reported a significant correlation between ETT movement and changes in cuff pressure [15]. In shoulder surgery performed in the beach chair position, some case reports described serious postoperative laryngeal complications, such as prolonged hoarseness, attributed to increased intracuff pressure [1,2]. Sim et al. reported a case of postoperative hoarseness due to arytenoid cartilage dislocation [2]. Other case reports have documented isolated neuropraxia [16] and combined recurrent laryngeal and hypoglossal nerve palsy, known as Tapia’s syndrome [17]. This rare and severe complication results in ipsilateral vocal cord paralysis and unilateral tongue muscle paralysis, leading to hoarseness, unilateral soft palate paralysis, and tongue deviation toward the affected side [17]. It has been speculated that ETT movement associated with head and neck position changes can alter intracuff pressure and volume, particularly under general anesthesia when the muscular tone is reduced [16].

As a secondary finding, all nine patients demonstrated cuff overinflation > 30 cm H₂O following inflation via syringe and manual palpation. It has been well documented that excessive cuff pressures above 30 cm H₂O reduce tracheal mucosa blood flow, with near-complete cessation occurring at pressures of 50 cm H₂O [18]. Hyperinflation of the cuff can lead to the development of tracheal ischemia during the endotracheal intubation period. In an experimental model, lateral wall pressures as low as 18-25 mmHg caused tracheal tissue damage within 2-4 hours [19]. Maintaining cuff pressures within the safe range of 20-30 cm H₂O is essential to both protect the lungs from aspiration and prevent tracheal mucosa injury [20].

Because reducing cuff pressure using manometry has been shown to significantly improve postoperative laryngotracheal symptoms (p < 0.0001), with an approximate effect size of 0.50 and a cost of only $0.34 per case [21,22], anesthesia providers are encouraged to incorporate routine manometry in patients requiring endotracheal intubation.

Several studies have demonstrated that palpating the external balloon or relying on the disappearance of an audible leak are unreliable methods for assessing appropriate intracuff pressure, often resulting in overinflation [23,24]. Rosero et al. found that in 25 of 28 patients undergoing laparoscopic surgery, intracuff pressures exceeded 30 cm H₂O when a 10 mL syringe was used; notably, 50% of patients had pressures as high as 100 cm H₂O [23]. Similarly, Braz et al., in a cross-sectional study of 85 patients in the intensive care unit (ICU) and post-anesthesia care unit (PACU), reported cuff pressures >40 cm H₂O in 55% (17/31) of ICU patients and 45% (10/22) of PACU patients, even in the absence of nitrous oxide [24]. Furthermore, Sengupta et al. found that the experience level of anesthesia providers did not reduce the risk of cuff overinflation [25]. In our study, using the traditional syringe inflation method, six out of nine patients (67%) had intracuff pressures exceeding 50 cm H₂O. These findings are consistent with our recent publication on a larger cohort, in which high pressures >50 cm H₂O were observed in ≥20% of cases when manometry was not used. Notably, in that study, reducing pressure with manometry significantly decreased postoperative laryngotracheal symptoms, including sore throat, hoarseness, and dysphagia [22].

One limitation of our study is that we measured changes in pressure only with respect to patient positioning, not over time. Choi et al. observed significant increases in cuff pressure associated with large volumes of irrigation fluid during the first two to three hours of shoulder arthroscopy (p < 0.005), and recommended close monitoring of ETT cuff pressure [26]. Another limitation is that the observed standard deviation of the change in cuff pressure from supine to sitting was 11.1 cm H₂O (Table 2), which is notably higher than the 6.7 cm H₂O used in our statistical power analysis [13,14]. As a result, our 99% CI for the mean pressure in sitting position ranged from 21 to 46 cm H₂O, compared to the target of 30 cm H₂O. Therefore, although the average change may appear negligible, the wide variability suggests that cuff pressure should still be individually checked after repositioning. We also acknowledge two other major limitations. First, our small sample size limited our ability to analyze associations with demographic variables such as age. Second, we did not quantify the degree of recumbency in the beach chair position, which may explain the relatively minimal change in pressure observed. Because the surgical setup and positioning techniques can vary across surgeons, our findings may not be generalizable to all clinical settings.

## Conclusions

Our study found that changing the patient’s position from supine to beach chair had no significant effect on intracuff pressure. However, six out of nine patients exhibited excessively high initial cuff pressures (>30 cm H₂O) following inflation by syringe and manual pilot balloon palpation. This finding reinforces prior research recommending the routine use of a manometer to accurately measure and adjust cuff pressure after intubation, thereby reducing the risk of postoperative laryngeal complications.
